# Six genetically linked mutations in the CD36 gene significantly delay the onset of Alzheimer's disease

**DOI:** 10.1038/s41598-022-15299-z

**Published:** 2022-06-29

**Authors:** Omar Šerý, Tomáš Zeman, Kateřina Sheardová, Martin Vyhnálek, Hana Marková, Jan Laczó, Jan Lochman, Petr Kralik, Kamila Vrzalová, Radka Dziedzinska, Vladimir J. Balcar, Jakub Hort

**Affiliations:** 1 Laboratory of Neurobiology and Pathological Physiology, Institute of Animal Physiology and Genetics, Czech Academy of Sciences, Veveří 97, 602 00 Brno 2, Czech Republic; 2 Laboratory of Neurobiology and Molecular Psychiatry, Department of Biochemistry, Faculty of Science, Masaryk University, Brno, Czech Republic; 3 International Clinical Research Center, St. Anne’s University Hospital Brno, Brno, Czech Republic; 4 1st Neurology Department, St. Anne’s University Hospital Brno, Brno, Czech Republic; 5 Memory Clinic, Department of Neurology, Charles University, Second Faculty of Medicine and Motol University Hospital, Prague, Czech Republic; 6 Neuroscience Theme, School of Medical Sciences, Faculty of Medicine and Health, The University of Sydney, Sydney, NSW Australia

**Keywords:** Molecular biology, Neuroscience, Biomarkers, Diseases, Medical research, Neurology, Pathogenesis, Risk factors

## Abstract

The risk of Alzheimer’s disease (AD) has a strong genetic component, also in the case of late-onset AD (LOAD). Attempts to sequence whole genome in large populations of subjects have identified only a few mutations common to most of the patients with AD. Targeting smaller well-characterized groups of subjects where specific genetic variations in selected genes could be related to precisely defined psychological traits typical of dementia is needed to better understand the heritability of AD. More than one thousand participants, categorized according to cognitive deficits, were assessed using 14 psychometric tests evaluating performance in five cognitive domains (attention/working memory, memory, language, executive functions, visuospatial functions). CD36 was selected as a gene previously shown to be implicated in the etiology of AD. A total of 174 polymorphisms were tested for associations with cognition-related traits and other AD-relevant data using the next generation sequencing. Several associations between single nucleotide polymorphisms (SNP’s) and the cognitive deficits have been found (rs12667404 with language performance, rs3211827 and rs41272372 with executive functions, rs137984792 with visuospatial performance). The most prominent association was found between a group of genotypes in six genetically linked and the age at which the AD patients presented with, or developed, a full-blown dementia. The identified alleles appear to be associated with a delay in the onset of LOAD. In silico studies suggested that the SNP’s alter the expression of CD36 thus potentially affecting CD36-related neuroinflammation and other molecular and cellular mechanisms known to be involved in the neuronal loss leading to AD. The main outcome of the study is an identification of a set of six new mutations apparently conferring a distinct protection against AD and delaying the onset by about 8 years. Additional mutations in CD36 associated with certain traits characteristic of the cognitive decline in AD have also been found.

## Introduction

Dementia as such is characterized by a decline of cognitive functions to the extent that it significantly impacts on activities of daily living. Dementia syndrome is mainly caused by underlying neurodegenerative diseases; the most common among these is Alzheimer's disease (AD), followed by dementia with Lewy bodies (DLB), Parkinson's disease dementia (PDD) and frontotemporal lobar degeneration (FTLD)^1–3^. Strictly speaking, conclusive diagnoses of the dementias can only be done using the presence in brain of characteristic histological features such as amyloid beta (Aβ) plaques and neurofibrillary tangles in AD, Lewy bodies in DLB and PDD and other formations (usually produced by aggregated or misfolded proteins) all of which can only be observed postmortem. As the present study is based on living subjects without histopathological confirmation of the disease, we are using an alternative designation such as “dementia of the Alzheimer type” (DAT) to reflect this fact^4^. In the following, we shall be using DAT, EOAD or LOAD (see below) in specific contexts but revert to AD in more general discourse.

Some cases of AD, particularly those diagnosed in patients under 65 years of age (early onset AD; EOAD), can be causally linked to specific mutations in a small number of genes^5^. In contrast, the late onset (age > 65) AD (LOAD), accounting for a great majority of AD cases, has both environmental and genetic causes. The heritable component of LOAD may be strong, estimated to be as much as 75%^6^ but its genetic substrate is poorly understood because of its complexity and a presumed interplay of many polymorphisms^7^. Several causal polymorphisms have been identified; some influence the age of onset^8,9^, others are linked to the rate of cognitive decline^10^ or to a specific cognitive profile^11^. Genetic polymorphism of Apolipoprotein E (ApoE), a cholesterol and lipid transporter, represents one of the strongest risk factors for LOAD^12^. Presence of one or two ApoE4 alleles implies a greater risk of LOAD, lower age of onset, more severe course of disease and poorer treatment response^13,14^. In addition to ApoE4, several other polymorphisms in various candidate genes have recently been identified as associated with LOAD^15–21^. The present study focuses on the polymorphisms of the candidate gene CD36 (“cluster of differentiation” 36). The product of the CD36 gene is a glycoprotein involved in lipid metabolism and trafficking^22,23^ both of which are key mechanisms contributing to the neurodegeneration in AD^24–26^ thus making CD36 an eminent molecule of interest when searching for mechanisms of the dementia-related neuronal loss.

Results of recent studies on CD36 gene polymorphisms (for a review see^23^) have shown that CD36 polymorphisms influence lipid metabolism and serum cholesterol level^27–29^, affect thresholds for orosensory detection of fatty acids^30–34^, are associated with the risk of obesity^30,31,33^ and cardiovascular disease^35^, correlate with the left ventricular mass volume^36^ and have even been associated with an aspect of intraocular pressure regulation^37^.

As a component of the plasma membrane and a type B scavenger receptor, CD36 is present in many types of cells including microvascular epithelium, phagocytes, dendritic cells, microglia, retinal cells, hepatocytes, cardiomyocytes as well others^38^, partaking, amongst other things, in the uptake of long chain fatty acids^39^ and oxidized lipoproteins^40^. It has been studied in relation to chronic ischemia and stroke as it is also a receptor for pathological ligands generated by high-fat diets and in fatty acid uptake, thereby additionally contributing to endothelial cell dysfunction^41^.

In its role as a scavenger receptor, CD36 acts by binding hydrophobic amyloid fibrils found in the AD^42^ and thus it is directly related to Aβ clearance by microglial phagocytosis. Moreover, interrupting the interaction between CD36 and Aβ compromises the activation of microglia (review^23^). This puts CD36 at a junction thought to be crucial in the cascade leading to the loss of neurons in AD; the rate of amyloid clearance may be decisive for the formation of amyloid plaques^43^. Furthermore, in animal models of AD, binding of Aβ to CD36 has been shown to be related to pro-inflammatory response; expression of microglial CD36 receptor, while enabling the interaction of microglia with amyloid fibrils, induces the release of proinflammatory cytokines (review^23^). Taken together, any changes in CD36 and/or CD36-related mechanisms could, therefore, have profound effects on how the brain functions. In fact, Abumrad et al.^44^ showed that CD36-deficient mice had an impairment in learning ability; from the perspective of the recent molecular studies mentioned above^42,43,45^ (for a review see^20^) such findings could be taken as an early hint that changes in the expression of CD36 may, indeed, contribute to the pathophysiology of dementia, including that of AD.

Better understanding of CD36 involvement in dementia-related mechanisms could lead to novel therapies. Potential approaches could aim either at inactivating CD36 with monoclonal antibodies, or, at the development of small molecules to control inflammation; alternatively, upregulation of CD36 to induce an increased amyloid β clearance could be considered^42,46^. The immunoinactivation of CD36 and its simultaneous upregulation would, however, potentially contradict each other; some balancing, centered on microglia, would be required. Evaluation of CD36 in AD clinical studies remains at a very early stage with limited knowledge about the impact of long chain fatty acids on AD progression and CD36 expression^42^.

Additionally, CD36 could play a role in the pathophysiology of some non-AD dementias: as a scavenger receptor it regulates microglial uptake of aggregated human α-synuclein which is a key component of Lewy bodies characteristic of Parkinson’s disease (PD) and the related dementias mentioned above^47^. To our knowledge, the role of CD36 in frontotemporal dementia has not been systematically investigated (see also below in “Discussion”).

The entire CD36 gene sequence in a well-defined longitudinal cohort of cognitively impaired subjects^48^ was analyzed by next generation sequencing (NGS) in the presented study. In addition to standard demographic parameters such as age, sex, body size or education, and detailed medical histories, a broad neuropsychological profile of each participant was compiled and included into the analysis. Specifically, the data were collected from subjects with Dementia of the Alzheimer type (DAT), mild cognitive impairment (MCI) and subjective cognitive decline (SCD) as well as a group of healthy older adults who served as controls. Subjects in each group underwent neuropsychological assessment using a range of tests (14 in total) designed to evaluate and quantify individual performance in five specific cognitive domains. We believe that such an approach—detailed analyses of particular psychological traits and/or characteristics of the disease on a background of genetic variants using well-defined population samples—may help to identify novel relationships between genotypes and phenotypes which might have, to date, been obscured in studies using very large and diverse groups of subjects^49^.

## Results

### Participants’ demography and neuropsychological assessment

The groups differed in all demographical variables and results of cognitive tests. In terms of cognitive performance, there is a gradient from subjects with normal cognition (HC and SCD) to the patients with both subtypes of MCI to those with DAT. Descriptive statistics for the whole group are shown in Table 1.

**Table 1:** Descriptive statistics.

	HC		SCD		naMCI		aMCI		DAT		P values
	N	Mean ± SD	N	Mean ± SD	N	Mean ± SD	N	Mean ± SD	N	Mean ± SD	
AGE (years)	51	69.18 ± 7.47	228	66.81 ± 7.86	90	69.26 ± 6.78	386	72.84 ± 7.51	250	74.26 ± 7.88	< 0.0001
Male	28	7 (25%)	189	62 (33%)	90	24 (27%)	281	128 (46%)	144	66 (46%)	0.0008
Female	28	21 (75%)	189	127 (67%)	90	66 (73%)	281	153 (54%)	144	78 (54%)	0.0008
Education (years)	26	16.04 ± 1.84	188	15.3 ± 2.95	90	13.58 ± 3.1	188	14.44 ± 3.13	101	13.82 ± 2.91	< 0.0001
MMSE	45	28.16 ± 3.94	220	28.69 ± 1.66	90	27.53 ± 2.09	378	25.53 ± 3.25	239	20.46 ± 4.73	< 0.0001
MEM_SC	38	0 ± 0.67	217	− 0.25 ± 0.64	87	− 0.49 ± 0.77	321	− 1.79 ± 0.96	199	− 0.89 ± 0.85	–^1^
MEM_DIF	0		119	0.14 ± 0.27	49	0.13 ± 0.3	62	0.12 ± 0.5	1	0.07	–^1^
EF_SC	37	0 ± 0.75	221	− 0.19 ± 0.97	90	− 1.27 ± 1.21	334	− 1.64 ± 1.49	161	− 2.68 ± 1.86	< 0.0001
EF_DIF	0		121	0.1 ± 0.37	49	0.16 ± 0.53	71	− 0.08 ± 1.22	1	1.5	0.037
LG_SC	36	0 ± 0.48	214	− 0.19 ± 0.49	89	− 0.65 ± 0.52	330	− 0.94 ± 0.67	182	− 1.56 ± 0.73	< 0.0001
LG_DIF	0		120	0.05 ± 0.22	48	0.08 ± 0.23	74	− 0.07 ± 0.33	1	0.4	0.0105
VS_SC	33	0 ± 0.52	213	− 0.19 ± 0.78	89	− 0.71 ± 0.92	302	− 1.32 ± 1.66	165	− 3.25 ± 2.18	< 0.0001
VS_DIF	0		119	0.93 ± 10.99	49	0.04 ± 0.59	72	− 0.28 ± 1.05	1	0.55	0.1226
AWM_SC	33	0 ± 0.67	220	− 0.12 ± 0.79	89	− 1.11 ± 0.74	348	− 1.16 ± 0.93	205	− 1.86 ± 1.15	< 0.0001
AWM_DIF	0		121	0.01 ± 0.36	49	0.12 ± 0.49	74	− 0.05 ± 0.54	1	0.48	0.1806
Body height (cm)	25	166.92 ± 7.34	182	169.3 ± 10.71	80	167.03 ± 7.59	188	168.47 ± 9.61	79	169.48 ± 10.18	0.2646
Body weight (kg)	25	74.68 ± 11.81	181	76.1 ± 15.83	80	80 ± 58.88	188	75.26 ± 14.44	79	72.41 ± 13.36	0.5499
BMI	25	26.75 ± 3.63	181	27.29 ± 15.64	80	28.76 ± 21.91	188	26.43 ± 4.19	79	25.13 ± 3.67	0.1238
Hypertension	33	19 (58%)	169	84 (50%)	66	31 (47%)	217	111 (51%)	105	56 (53%)	0.8577
Hypercholesterolemia	31	14 (45%)	159	79 (50%)	57	29 (51%)	207	77 (37%)	101	41 (41%)	0.1116
CAD	30	1 (3%)	162	8 (5%)	62	3 (5%)	206	19 (9%)	99	14 (14%)	0.0746
Congestive heart failure	31	0 (0%)	164	2 (1%)	63	3 (5%)	213	7 (3%)	91	4 (4%)	0.3453
CLTI	30	0 (0%)	149	11 (7%)	56	1 (2%)	203	11 (5%)	92	7 (8%)	0.3446
Diabetes	31	1 (3%)	163	15 (9%)	62	9 (15%)	216	28 (13%)	94	23 (24%)	0.0063

^1^z-scores are not comparable between groups because different cognitive tests were used (see Table 5 for details).

*SD* standard deviation, *BMI* body mass index, *CAD* coronary artery disease, *CLTI* chronic limb-threatening ischemia, *DAT* dementia of the Alzheimer's type, *MEM_SC* the memory domain residual z-score at the first examination, *EF_SC* the executive functions domain residual z-score at the first examination, *LG_SC* the language domain residual z-score at the first examination, *VS_SC* the visuospatial memory domain residual z-score at the first examination, *AWM_SC* the attention and working memory domain residual z-score at the first examination, *MEM_DIF* average increment of MEM_SC per year during the 2 years since the first examination (intercept of the OLS regression line), *EF_DIF* average increment of EF_SC per year during the 2 years since the first examination (intercept of the OLS regression line), *LG_DIF* average increment of LG_SC per year during the 2 years since the first examination (intercept of the OLS regression line), *VS_DIF* average increment of VS_SC per year during the 2 years since the first examination (intercept of the OLS regression line), *AWM_DIF* average increment of AWM_SC per year during the 2 years since the first examination (intercept of the OLS regression line).

### Results of NGS sequencing

**Figure 1:**
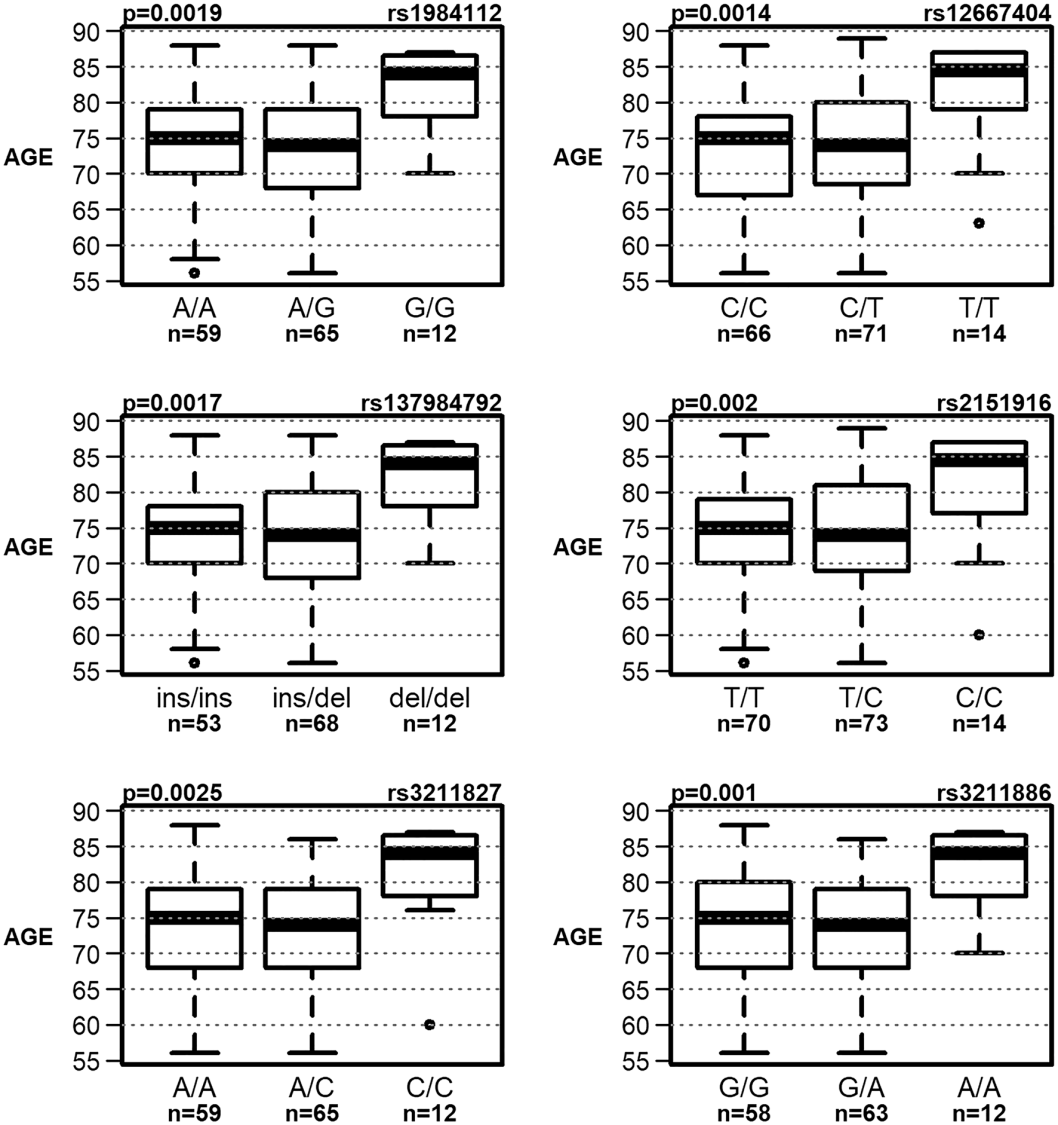
Association of polymorphisms with AGE in dementia of the Alzheimer's type (DAT) group (statistically significant at a significance level of 0.1 after correction for multiple comparisons).

**Figure 2:**
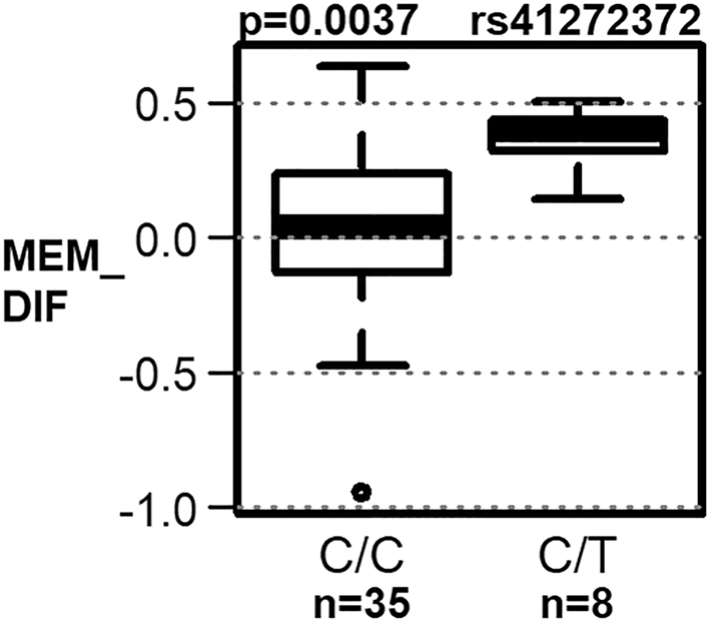
Association of polymorphism rs41272372 with MEM_DIF (average increase or decrease of the memory domain residual z-score in 2 years after the first examination) in naMCI group (statistically significant at a significance level of 0.1 after correction for multiple comparisons).

**Figure 3:**
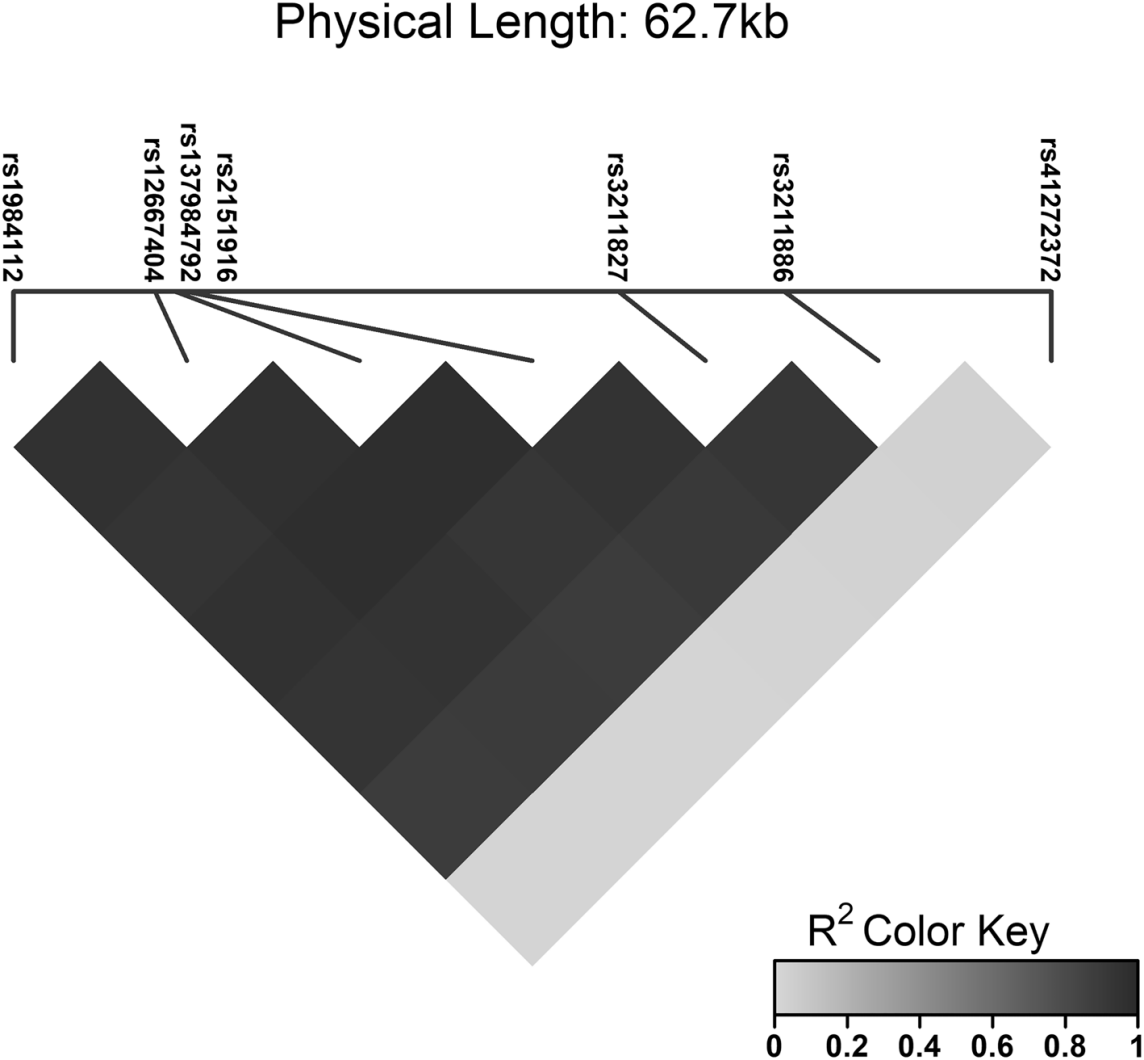
Linkage disequilibrium (R^2^) for identified polymorphisms in the gene for CD36.

The entire CD36 gene was sequenced using the NGS method between positions 80,602,188 and 80,679,277 according to the genome assembly GRCh38 (NCBI). This netted a total of 174 polymorphic sites. Of these 174 polymorphisms, after eliminating the effects of multiple comparisons, a total of seven polymorphisms remained, which are statistically significantly related to the tested variables (Table 2). Of these seven polymorphisms, six polymorphisms (rs1984112, rs12667404, rs137984792, rs2151916, rs3211827 and rs3211886) were related to the age of onset of Alzheimer's disease (termed “AGE” in tables and figures). Genotypes GG (rs1984112), TT (rs12667404), del/del (rs137984792), CC (rs2151916), CC (rs3211827) and AA (rs3211886) were associated with the onset of DAT at higher age; the difference was highly statistically significant and amounted to about 8 years (Fig. 1). In the group of SCD patients, genotype CC (rs12667404) was associated with worse performance in the language domain and genotype AA (rs3211827) with the greater decline of executive functions. In the group of naMCI patients, ins/del genotype (rs137984792) was associated with worse visuospatial performance and genotype CC (rs41272372) with worse performance in executive function, and memory domains (Fig. 2). Noticeably, linkage equilibrium analysis indicated (Fig. 3) that all six polymorphisms associated with the age of onset of DAT are in a very strong linkage. Comparisons of allelic frequencies between our studied groups and the frequencies listed in Genome Aggregation Database (GnomAD^50^) for the European population did not show any significant differences (Table 3).

**Table 2:** Association of selected polymorphism with used variables.

SNP	Position/region	Group	Variable	Genotype 1	Genotype 2	Genotype 3	P value
rs1984112	80,613,604/intron	DAT	AGE	A/A 74.05 ± 7.58 (n = 59)	A/G 73.31 ± 8 (n = 65)	G/G 81.92 ± 5.57 (n = 12)	0.0019^+^
rs12667404	80,622,147/intron	DAT	AGE	C/C 73.17 ± 7.81 (n = 66)	C/T 73.55 ± 8.08 (n = 71)	T/T 81.36 ± 7.32 (n = 14)	0.0014^+^
		SCD	LG_DIF	C/C 0 ± 0.21 (n = 43)	C/T 0.1 ± 0.24 (n = 39)	T/T 0.05 ± 0.2 (n = 9)	0.0468
rs137984792	80,623,351/intron	DAT	AGE	ins/ins 73.83 ± 7.74 (n = 53)	ins/del 73.04 ± 8.06 (n = 68)	del/del 81.92 ± 5.57 (n = 12)	0.0017^+^
		naMCI	VS_SC	ins/ins − 0.48 ± 0.88 (n = 27)	ins/del − 1.01 ± 0.86 (n = 25)	del/del − 0.76 ± 0.93 (n = 11)	0.0347
rs2151916	80,624,067/intron	DAT	AGE	T/T 73.41 ± 7.33 (n = 70)	T/C 73.86 ± 8.3 (n = 73)	C/C 81 ± 7.98 (n = 14)	0.002^+^
rs3211827	80,650,144/intron	DAT	AGE	A/A 73.44 ± 8.22 (n = 59)	A/C 72.85 ± 7.88 (n = 65)	C/C 81.08 ± 7.81 (n = 12)	0.0025^+^
		SCD	EF_SC	A/A − 0.36 ± 1.26 (n = 71)	A/C 0.09 ± 0.71 (n = 63)	C/C 0.33 ± 0.48 (n = 11)	0.034
rs3211886	80,660,123/intron	DAT	AGE	G/G 73.76 ± 8.05 (n = 58)	G/A 72.78 ± 7.69 (n = 63)	A/A 81.92 ± 5.57 (n = 12)	0.001*
rs41272372	80,676,256/3′UTR	naMCI	MEM_DIF	C/C 0.05 ± 0.31 (n = 35)	C/T 0.37 ± 0.11 (n = 8)		0.0037^+^
		naMCI	EF_SC	C/C − 1.43 ± 1.25 (n = 60)	C/T − 0.67 ± 0.82 (n = 11)		0.0388

Only those polymorphisms whose association with any of the variables was statistically significant at a significance level of 0.1 after correction for multiple comparisons are reported. For these, the mean values ± standard deviations of the variables for which P values were lower than 0.05 are given.

*SCD* subjective cognitive decline, *naMCI* non-amnestic mild cognitive impairment, *DAT* dementia of the Alzheimer's type, *AGE* the age at the first examination in years, *EF_SC* the domain of the executive function residual z-score at the first examination, *LG_SC* the language domain residual z-score at the first examination, *MEM_SC* the memory domain residual z-score at the first examination, *VS_SC* the visuospatial memory domain residual z-score at the first examination, *MEM_DIF* average increment of MEM_SC per year during the 2 years since the first examination (intercept of the OLS regression line), *LG_DIF* average increment of LG_SC per year during the 2 years since the first examination (intercept of the OLS regression line).

*P values significant at 0.05 after correction for multiple comparisons, ^+^P values significant at 0.1 after correction for multiple comparisons, n—sample size.

**Table 3:** Comparisons of allelic frequencies between our study group and the frequencies listed in Genome Aggregation Database (GnomAD^50^) for the European population.

Polymorphism	Allele/variant	Current data allele frequency	GnomAD allele frequency
rs1984112	G	0.33	0.34
rs12667404	T	0.32	0.34
rs137984792	TGTG	0.35	0.34
rs2151916	C	0.32	0.34
rs3211827	C	0.31	0.34
rs3211886	C	0.33	0.33
rs41272372	T	0.08	0.10

### In silico search for potential transcription factor binding sites

**Figure 4:**
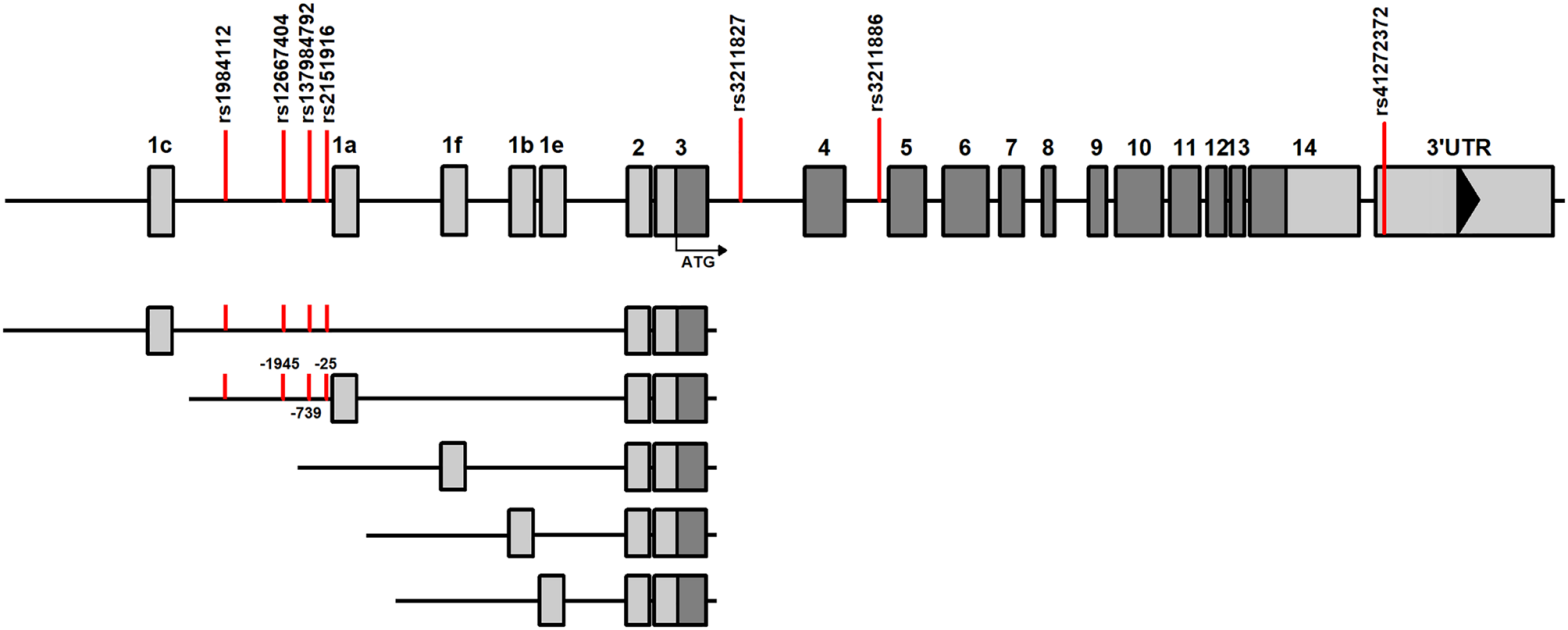
Position of 7 polymorphisms in the gene for CD36 and exons and different 5′ untranslated region of CD36 gene. Light shadow boxes represent untranslated mRNA sequence, the dark shadow box represents the translated sequence. The positions of identified CD36 polymorphisms are marked by red vertical lines. Overview of the 5′ untranslated region of CD36. Exon 1a denotes the alternative first exon and the accession number [GenBank: NM_001371077]. Exon 1b denotes the alternative first exon and the accession number [GenBank: NM_000072]. Exon 1c denotes the alternative first exon and the accession number [GenBank: NM_001371075]. Exon 1e denotes the alternative first exon annotated on the sequence and the accession number [GenBank: NM_001289909].

The positions of seven important CD36 gene polymorphisms are shown in Fig. 4. Among the polymorphisms presently identified as significantly associated with a particular psychological trait and the age of onset of DAT, four are located in the first intron of the CD36 gene, one in the third intron, one in the fourth intron, and one in the 3′UTR region of the CD36 gene (Fig. 4). Notably, polymorphisms rs12667404, rs71518997 and rs2151916 are in the promoter region of the alternative mRNA isoform represented by exon variant 1a (Fig. 4). All three SNPs are in haplotype block represented by a high degree of linkage disequilibrium (Fig. 3, Table 4). Search for putative transcription factor binding sites (TFBSs) focused on sequences in promoter regions of the alternative first exon 1a represented by 2000 base pairs upstream. Analysis of rs12667404, rs71518997 and rs2151916 polymorphisms in four homozygous controls and patients indicated that their presence could influence characteristics of binding sites for transcription factors (Table 4). Allele T in SNP rs12667404 would cause a loss of binding sites for transcription factors NF-kappaB1 and RXR-alpha and could result in an appearance of a new binding site for TF TFII-I (Table 4); the allele T is also significantly associated with the age of onset in DAT group. Allele C of rs2151916 polymorphism was reported to cause a loss of binding site for transcription factor C/EBPbeta and GR-beta. Additionally, the deletion in rs71518997 polymorphism leads to the loss of binding site for transcription factor SRY and the occurrence of new binding site for transcription factor NF-1 (Table 4).

**Table 4:** Sequence variants in the CD36 5′ regulatory region of patients with Alzheimer’s disease.

Variation^1^ and polymorphism	TFBS alterations^2^ (dissimilarity/RE equally)	Association of polymorphisms with AGE in DAT group
− 1945 (C > T) rs12667404	− NF-kappaB1 [T00593] (6.19/0.04) − RXR-alpha [T01345] (6.97/0.37) + TFII-I [T00824] (6.58/0.98)	Yes
− 739 (delTG) rs71518997	− SRY [T00997] (7.18/0.31) + NF-1 [T00539] (8.79/0.24)	Yes
− 25 (T > C) rs2151916	− GR-beta [T01920] (4.20/7.81) − C/EBPbeta [T00581] (1.37/15.63)	Yes

^1^Positions relative to the CD36 Exon 1a TSS [Gen-Bank: DA741325] at position 80,624,092 in NCBI reference sequence NC_000007.14.

^2^PROMO analysis using annotations in TRANSFAC version 8.3 entries.

### miRNAs binding sites

Besides, using a miRBase of known mature human micro RNA’s (miRNA’s)^51^ we identified possible binding sites in the vicinity of two polymorphisms, rs1984112 and rs3211827 as putative targets for specific miRNAs. In the presence of allele G of polymorphism rs1984112, there was a possible weaker interaction with hsa-miR-6529-3p and loss of interaction with hsa-miR-4496 (Table 5). Within rs3211827 polymorphism, two possible candidate miRNA (hsa-miR-2681-5p and hsa-miR-329-5p) for an allele A and one candidate miRNA (hsa-miR-6808-5p) for allele C were found (Table 5). However, further analysis by the miRNA target detection algorithm RNA22 v2^52^ indicated that the likelihood of a specific interaction of the mi-RNA with the putative target in the locus is quite small.

**Table 5:** Analysis of the significant polymorphisms for similarity with miRBase of mature miRNAs^51^ specific to human by using BLASTN search algorithm.

Polymorphism	Allele	ID miRNA	Strand	Score	E-value
rs1984112	A	hsa-miR-6529-3p	−	73	1.1
		hsa-miR-4496	+	64	6.0
	G	hsa-miR-6529-3p	−	64	5.7
rs3211827	C	hsa-miR-6808-5p	+	62	8.7
	A	hsa-miR-2681-5p	−	68	2.8
		hsa-miR-329-5p	−	64	6.0

## Discussion

The overall outcome of extensive neuropsychological investigations (14 tests related to four specific psychological domains; all selected as relevant in the development and progression of DAT) indicated a significant association between genotype CC (rs12667404) and impaired performance in the language domain and between genotype AA (rs3211827) and a greater decline of executive functions in the SCD subjects. In the naMCI subjects, ins/del genotype (rs137984792) was associated with impaired visuospatial performance and genotype CC (rs41272372) with disturbing the performance in executive functions and memory domains (Fig. 2).

The strong association between the age of onset of DAT and several of the polymorphisms has emerged as, arguably, the most significant outcome of the present study. This refers to the genotypes GG (rs1984112), TT (rs12667404), del/del (rs137984792), CC (rs2151916), CC (rs3211827) and AA (rs3211886). The patients with these genotypes (six out of the total of 174 polymorphisms found in CD36 gene) were, when diagnosed with DAT, on average 8 years older, than the rest of the DAT cohort. Moreover, the two exceptions had the ApoE4 allele, which would have, almost certainly, nullified the CD36 effects (for the sample as a whole, however, we excluded any systematic effect of ApoE4 allele on the present results; data not shown). While there are various possible interpretations of this observation, the most parsimonious conclusion would seem to be that the DAT-onset associated CD36 polymorphisms may offer a (limited) protection against the disease by significantly delaying the appearance of dementia symptoms. Could the effect be explained on a cellular and molecular basis?

Recently, several alternative mRNA isoforms transcribed from the first exons of the CD36 gene have been described^53,54^. The existence of tissue-specific expression patterns of the alternative first exons of CD36 suggests that the alternative forms of the first exon of CD36 are regulated individually and tissue-specifically^53^. Polymorphisms rs12667404, rs71518997 and rs2151916 related to the later onset of DAT occur in the promoter region of the alternative mRNA isoform represented by exon variant 1a (Fig. 4). The strong genetic linkage of the polymorphisms makes it harder to pinpoint which polymorphism(s) and/or molecular mechanism(s) is/are most likely to be decisive in timing the onset of DAT. Therefore, we turned to in silico approach to seek more specific interpretations of the data.

In silico search for putative transcription factor binding sites (TFBSs) sequences indicated that the polymorphisms were within the sequences of TFBSs and could, therefore, change/abolish their affinities for the transcription factors and, consequently, influence the expression of CD36; allele T in SNP rs12667404 results in the loss of binding sites for transcription factors NF-kappaB1 and RXR-alpha but forms a new binding site for TF TFII-I (Table 5). This may be directly relevant to the interpretation of the present data; Yamanaka et al.^55^ described RXR-alpha as a functionally relevant transcription factor for elevated Aβ phagocytosis by CD36 in primary microglia and the loss of the binding site in patients with delayed DAT could lead to the change in CD36 expression that could negatively influence Aβ phagocytosis by microglia but, perhaps more significantly, it could diminish production of proinflammatory cytokines (see below). This would be consistent with a slower development and or delay in the onset of DAT. However, SNP rs12667404 position at some distance from the starting codon could raise doubts as to whether such a simple change located so far away from the transcribed sequences could contribute much to the delayed onset of DAT. In contrast, the other two SNPs showing a significant association with the age of DAT onset are located nearer the transcribed sequences and thus their role in the regulation of transcription of alternative mRNA exon variant 1a appears more plausible. Based on the location of the polymorphisms, and on the presence of another binding sequence in the vicinity, one of the more probable candidates is SNP rs2151916 located 25 bp downstream of the relative CD36 Exon 1a transcription starting site (TSS) in which the presence of allele C results in the loss of binding site for transcription factor C/EBPbeta which has been reported as having a role in transcriptional regulation of the gene CD36^56,57^. C/EBPbeta can up-regulate CD36 gene transcription through a C/EBP-responding element at the proximal promoter^57^. Similarly, the occurrence of C allele in rs2151916 leads to the loss of binding of transcription factor GR-beta. Deletion in rs71518997 polymorphism leads to the loss of binding site for SRY and to an appearance of a new binding site for NF-1 transcription factor which could also be important in the regulation of CD36 gene expression.

In the case of rs1984112 and rs3211827 polymorphisms, in silico analyses initially indicated possible involvement of putative miRNAs binding sites. More detailed evaluations made the plausibility of such interpretations questionable; in contrast to the earlier-mentioned polymorphisms, both rs1984112 and rs3211827 are found in intronic regions which seem to be devoid of any regulatory features. However, a very strong linkage of the rs1984112 and rs3211827 with other three polymorphisms in the promoter region of alternative CD36 mRNA isoform in question may explain their role in the regulation of CD36 expression (Fig. 3). Overall, the analyses performed in silico provide us with a basis to discuss, at least in general terms, the potential role(s) of CD36 in the pathophysiology of AD and offer plausible explanations for the present observations.

**Figure 5:**
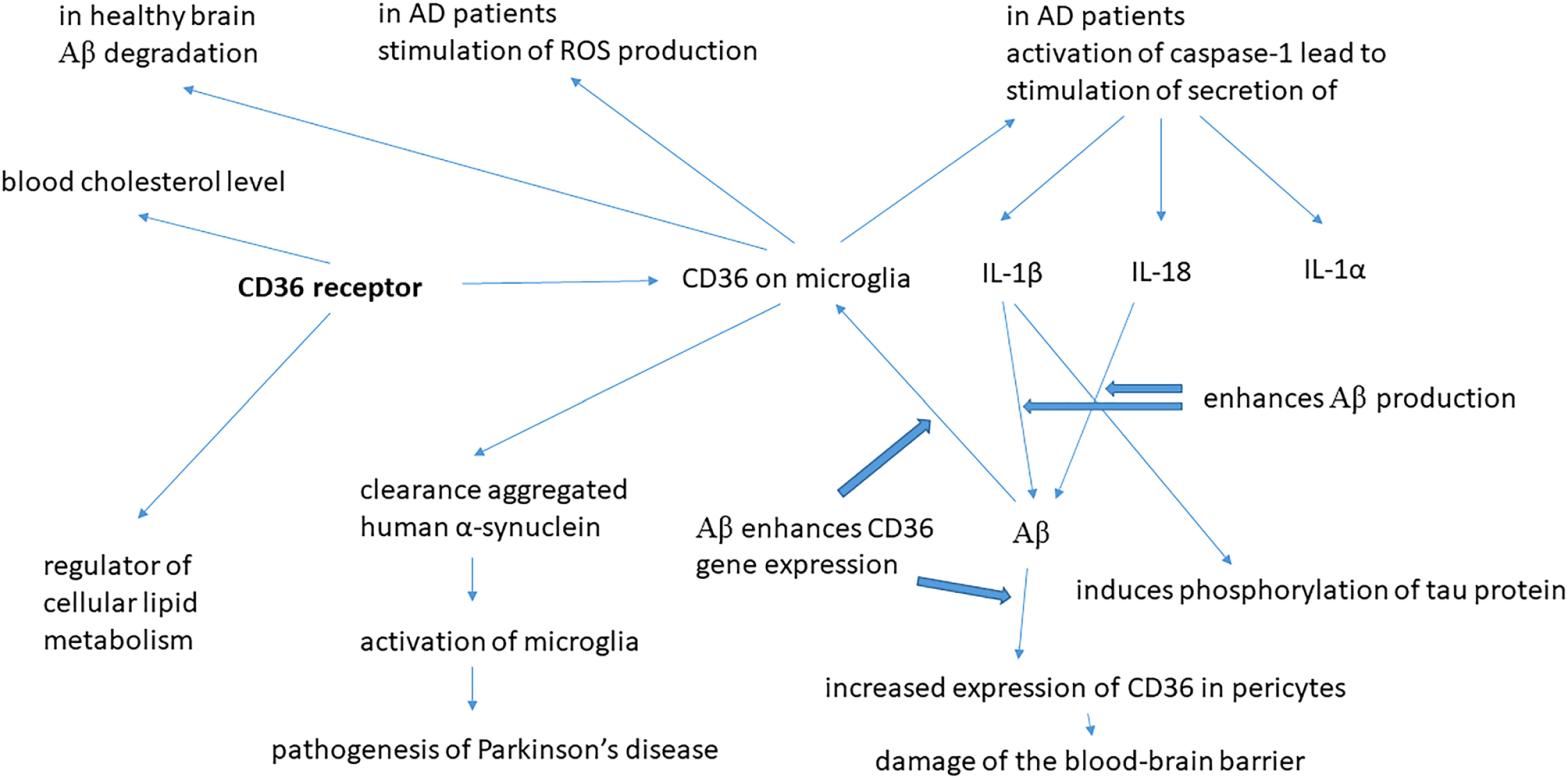
Some roles of the CD36 receptor in the pathogenesis of Alzheimer's disease and Parkinson’s disease.

As mentioned earlier, altered lipid metabolism (particularly with respect to the regulation of cholesterol and the movement of fatty acids), formation of neurofibrillary tangles, Aβ accumulation (followed by the deposition of amyloid plaques in extracellular space) and the excessive production of ROS (reactive oxygen species) are among the most important putative etiologies of AD^23,25,58–60^. The most striking feature of CD36 biochemistry is the way in how several of the mechanisms in which CD36 is involved—regulating cholesterol, transport of fatty acids, production of ROS, fostering an inflammatory process and interaction with Aβ—neatly fit at least three of the AD mechanisms mentioned above. CD36 may have the following three specific roles acting separately, yet, potentially, in synergy, all contributing to the pathogenesis of AD. (1) In the early stages of AD, Aβ accumulation induces CD36 expression in microglia/macrophages and that could help to reduce, via negative feedback, the Aβ deposits^61^; in this role, CD36 would serve as early protection against the formation of Aβ plaques. (2) CD36 promotes neurodegeneration by stimulating ROS production^62^; increasing Aβ deposits would, at this (later) stage of AD, continue to stimulate the CD36 expression, leading to excessive ROS production, directly killing cells, vulnerable neurons in particular^61^. (3) CD36 stimulates the production of inflammatory cytokines; CD36 cooperates with toll-like receptor (TLR) heterodimer of TLR4-TLR6 to recognize oxLDL and fibrillary Aβ; inducing an inflammatory response. CD36 with bound Aβ activates cytosolic sensor NLRP3 (NOD-like receptor 3) and then NLRP3-inflammasome complex activating caspase-1, which can then catalyze the cleavage of pro-IL-1β and pro-IL-18 promoting secretion of IL-1β, IL-18 and IL-1α^63^. Inflammasomes, which cleave precursors of interleukin-1β (IL-1β) and IL-18 to generate their active forms, play an important role in the inflammatory response in AD pathogenesis^23,64^. The involvement of the CD36 gene in the pathophysiology of Alzheimer's disease is summarized in Fig. 5.

In animal experiments, Zhang et al.^65^ found that a knockout of CD36 resulted in an anxious phenotype and more aggressive behaviour. Balkaya et al.^66^ studied CD36 knockout in a mouse model of stroke and found that the lack of CD36 helps in the learning and relearning during the recovery from the stroke. Abumrad et al.^44^ reported that CD36 deficient mice displayed several normal patterns of activity but appeared to have a significant impairment in learning ability. The different conclusions of these experiments may reflect different experimental settings, and may not be directly applicable to the present discussion, e.g. the stroke model involves a sudden loss of neurons, very different from that in the chronic neurodegenerative disease such as the human LOAD. The benefit from the lack of CD36 that helps in the learning and relearning during the recovery from the stroke observed by Balkaya et al.^66^, could be related to our results. We would have to assume that CD36 has little if any, the protective effect at the earliest, prodromal stages of AD while reduced expression of CD36 possibly associated with the set of polymorphisms/mutations found in the present study could, perhaps, cause a shift the onset of Alzheimer's disease to a significantly later age (see the next paragraph).

We have also noticed that, in the group of SCD patients, the genotype CC (rs12667404) was associated with a lower performance in the language domain, while being, at the same time, associated with the delayed onset of DAT. We wonder whether these—apparently contradictory—observations might not reflect a lower CD36 expression associated with the genotype. The lower expression of CD36 would negatively influence Aβ phagocytosis by microglia at the earliest stage of dementia (thus promoting a dementia-related mechanism and contributing to SCD) but would provide a degree of protection during the later stages of the pathological process by mitigating the CD36-dependent inflammatory mechanisms and delaying the appearance of the fully blown DAT.

Polymorphisms rs137984792 and rs41272372 found to be, in the naMCI subjects, associated with visuospatial and executive function and memory domains performance are placed in promotor and 3′ untranslated region of CD36 gene, respectively. The two polymorphisms are in a linkage disequilibrium with the above functional polymorphisms (Fig. 3), and this potentially implies (or, at least, it does not preclude) an effect on CD36 gene expression.

The above discussion notwithstanding, we do not wish to imply that the presently identified polymorphisms only influence the regulation of CD36 expression and, perhaps, alter the level of its expression in brain tissue. Nor is it necessary to postulate that the polymorphisms are effective solely or primarily via the involvement of CD36 in brain inflammatory cascade or by binding the amyloid fibrils in AD brains. For example, CD36 has been linked to cardiovascular diseases including stroke or chronic ischemia^42,66^ and cerebral microhemorrhages caused by ministrokes could contribute to the development of a DAT^67^, thus potentially mediating the effect of CD36 polymorphisms on AD. Taken together, there are diverse mechanisms, potentially involved in the etiology of AD, where CD36 has a role (recent reviews^42,68,69^). Results of very recent animal studies expand this diversity by demonstrating CD36 involvement in an AD-associated blood brain barrier disruption^70^, and in an interaction with the nerve growth regulator1 (NEGR1)^71^, i.e., in AD-related mechanisms distinct from those active in the amyloid/tau pathways and/or the amyloid-β phagocytosis by microglia. Conversely, another recent report implicated a DNA transcription regulator microRNA (miRNA-485-3p) in the CD36 mediated amyloid-β phagocytosis and even suggested it as a potential AD therapeutic agent^72^. Yet our in silico analyses found no potential interplay of miRNA-485-3p and any of the five AD-related polymorphisms identified by the present whole-gene sequencing study (Table 5), thus implying that not all AD-related regulatory sites in CD36 gene must necessarily be located next to (and be influenced by) AD-related SNP’s. Such observations illustrate the challenges in testing associations of specific genes with a particular disease and imply that further studies focusing on additional aspects of CD36 regulation and/or actions, need to be done before we can adequately understand its role(s) in the etiology of AD.

Could polymorphisms in CD36 gene influence the risk of other neurodegenerative diseases, in addition to AD (and, perhaps, multiple sclerosis^73^? As mentioned in “Introduction”, CD36 has been shown to be involved in the actions of extracellular aggregates of α-synuclein known to be formed in Parkinson’s disease and related dementias^47^. PD and associated disorders should, therefore, be included in future CD36 case association studies. If the effect of CD36 on the actions of protein aggregates prove to be more common and involve other protein aggregates in addition to those of α-synuclein, the studies should extend to a broader range of neurological diseases. These should include fronto-temporal dementia and the closely related motor neuron disease (amyotrophic lateral sclerosis), where protein aggregates also occur^74^, but the key mechanisms leading to the loss of neurons remain poorly understood despite the identification of several molecules of interest^75–78^.

## Conclusions

The present study employed detailed psychological characterization of patients with Alzheimer’s dementia and dementia-related conditions in combination with complete sequencing (NGS) of a selected “candidate” gene (CD36). This approach has identified six genetically linked polymorphisms (SNP’s) which appear to have a decisive influence on the age of onset of Alzheimer’s disease. Furthermore, we have found two SNP’s associated with language and executive functions in patients with subjective cognitive decline as well as two SNP’s associated, respectively, with language and executive functions, both in the group of patients with non-amnesic mild cognitive impairment. The study was technically demanding, requiring individual administration of > 14K psychological tests, each necessitating direct involvement of specialized professional personnel i.e. psychiatrists or practicing psychologists (in addition to the whole gene sequencing) but it proved its value. The outcomes demonstrate that focusing on selected genes of interest in well-characterized groups of subjects can find unique genetic associations and help to unravel the heritability of mental disease.

## Methods

### Subjects

The study sample included 1005 subjects from memory clinic centers^48^—International Clinical Research Center at St. Anne’s University Hospital in Brno and Memory Clinic of Department of Neurology at Motol University Hospital in Prague. Patients with DAT (DAT group, n = 250), amnestic MCI (aMCI group, n = 386), non-amnestic MCI (naMCI group, n = 90) and SCD (SCD group, n = 228) were referred to the memory clinics by general practitioners, neurologists, and psychiatrists because of subjective cognitive complaints reported by themselves and/or by their close informants. Healthy older adults were recruited from the University of the Third Age at Masaryk University in Brno and from relatives of the patients and hospital staff; they served as a healthy control group (HC group, n = 51). All subjects underwent standard clinical and laboratory evaluations, brain magnetic resonance imaging (MRI), comprehensive patient history taking and neuropsychological examination at the start of the study and thereafter on annual (or biannual in SCD patients) basis. The number of observations for each subject depends on the year in which they were included in the study and, as a result, may vary from one to five. Non-demented subjects were followed up until they progressed to dementia. Follow-up procedures were identical to those done during the baseline visit. The participants with dementia syndrome were classified according to the type of dementia when admitted to the project using the initial (“baseline”) set of data and were not followed up, therefore only the baseline data were available for the analysis.

The subjects in DAT group met the current criteria for probable AD^79^, with an insidious onset of symptoms, showing an evident decline from previous levels of functioning not explained by delirium or any other major psychiatric disorder or neurological disease. Patients with aMCI and naMCI met the clinical criteria for MCI^80^, including subjective memory complaints, evidence of cognitive deficit on neuropsychological testing, generally intact activities of daily living, and absence of dementia. If there was an evidence of memory impairment, the patients were classified as aMCI; if the impairment was limited to non-memory domains (executive, language and/or visuospatial functions; Table 6), the patients were classified as naMCI. Both aMCI and naMCI groups included single- and multiple-domain phenotypes. Impairment in a cognitive domain was established if the subject scored more than 1.5 standard deviations (SD) below the mean of age- and education-adjusted norms on any test within the cognitive domain. Patients with SCD met the published criteria for SCD^81^, including self-reported persistent cognitive decline within the last 5 years in comparison to the previous level unrelated to an acute event with performance on standardized cognitive tests adjusted for relevant demographic variables within the normal range. HC did not report any subjective cognitive complaints requiring doctors’ attention as verified in a structured interview by an experienced neuropsychologist. Their cognitive performance on comprehensive neuropsychological assessment was within the normal range (that is, none of the scores were ≥ 1.5 SD below the mean of age- and education-adjusted norms). The diagnoses were determined at the joint meetings of neuropsychologists with neurologists and were based on mutual consent.

**Table 6:** Summary of neuropsychological tests according to cognitive domains.

Domain	Abbreviation	Neuropsychological tests
1. Attention and working memory	AWM	1.1 Digit Span Forward (DS-F)
		1.2 Digit Span Backward (DS-B)
		1.3 Trail Making Test (TMT) A
2. Memory	MEM	2.1 Logical Memory I, delayed recall after 20 min (LM-DR) 2.2 Rey Auditory Verbal Learning Test, sum of trial 1–5 (RAVLT 1–5)^1^ 2.3 RAVLT, delayed recall after 30 min (RAVLT-DR)^1^
		2.4 Enhanced Cued Recall, free recall (ECR-FR)^2^
		2.5 Enhanced Cued Recall, total recall (ECR-TR)^2^
3. Executive function	EF	3.1 TMT B
		3.2 Phonemic Verbal Fluency (P-VF), Czech version with letters N, K, P
4. Language	LG	4.1 Semantic Verbal Fluency (SV-F), Animals
		4.2 S-VF, Vegetables
		4.3 Boston Naming Test (BNT), 30 odd-items version
5. Visuospatial function	VS	5.1 Rey–Osterrieth Complex Figure Test (ROCFT), copy condition

^1^Tests that were administered in SCD and aMCI groups only, ^2^tests that were administered in DAT (dementia of the Alzheimer's type) group only.

Exclusion criteria were the following: age below 55, history or evidence of a neurological disease with a potential impact on cognition (stroke, traumatic brain injury, neuroinfection, etc.), history of psychiatric disease (major depressive disorder, bipolar affective disorder, generalized anxiety disorder, schizophrenia, etc.) and abnormal neurological signs, including gait or movement difficulties. Detailed inclusion and exclusion criteria have been described elsewhere^48^.

All subjects in this study signed a written informed consent that was approved by ethics committee of St. Anne’s University Hospital Brno and Motol University Hospital (Praha). The procedures were in accordance with the Helsinki Declaration of 1975 (revised 2000).

### Neuropsychological assessment

Following an assessment for their global cognitive function as measured by the Mini-Mental State Examination (MMSE test), all participants underwent a range of specific neuropsychological tests designed to evaluate their performance in the following cognitive domains: (1) attention and working memory (AWM), measured by the Forward and Backward Digit Span Subtests (DS-F, DS-B, respectively, from the Wechsler Adult Intelligence Scale—Revised), and the Trail Making Test (TMT) A; (2) memory (MEM), measured with the Logical Memory, delayed recall after 20 min, adaptation from the Uniform Data Set (UDS-cz 2.0), the Rey Auditory Verbal Learning Test, sum of trial 1 to 5 (RAVLT 1–5) and delayed recall after 30 min (RAVLT-DR), and the Enhanced Cued Recall, free and total recall (ECR-FR, ECR-TR, respectively); (3) executive function (EF) measured with the TMT B and the phonemic verbal fluency tests (P-VF, Czech version with letters N, K, P); (4) language (LG) measured with the Boston Naming Test (BNT), 30 odd-items version, and the semantic verbal fluency test (S-VF, animals and vegetables); and (5) visuospatial function (VS) measured with the Rey–Osterrieth Complex Figure Test (ROCFT), the copy condition and the Clock Drawing Test (CDT). Each cognitive domain was expressed as a composite domain score, calculated as the average of z-scores for each of the tests within the specific cognitive domain. The z-scores were calculated for each of the tests using the mean and the standard deviation of the values determined during the first examination of HC participants as a reference (z-scores for TMT A and B were multiplied by negative ones to express the values in the same direction as the other neuropsychological values). For the memory domain, the composite score partially differed between the SCD and aMCI groups and the DAT group. Since the RAVLT is too difficult to be administered in patients with dementia (leading to the floor effects), it was used only in patients with SCD and aMCI. In patients with dementia the ECR was used instead. The maximum time for completion of the TMT A and B were 150 s and 300 s, respectively, and those who were unable to complete it in a given time were scored as 151 s and 301 s, respectively. Table 6 summarizes the neuropsychological tests that were used to assess the cognitive domains.

### Next generation sequencing

Blood samples were collected by qualified hospital personnel; standard EDTA anticoagulant collection tubes were used. DNA from blood samples was isolated by Prepito NA Body Fluid Kit (chemagen, PerkinElmer) according to the manufacturer’s instructions. Concentration of DNA was measured using iQuant™ dsDNA HS Assay Kit (ABP Biosciences, Beltsville, MD, USA).

Library preparation was performed by SeqCap EZ System (Roche Sequencing Solutions, Pleasanton, CA, USA) with probes targeting 73 genes including CD36 according to the manufacturer’s instructions. The indexed paired-end libraries were prepared from 200 ng genomic DNA in 35 µl 10 mM Tris–HCl (pH 8.0) by enzymatic fragmentation using KAPA HyperPlus Library Preparation Kit, and KAPA Dual-Indexed Adapter Kit (both Roche). Amplified sample libraries were quantified using iQuant™ dsDNA HS Assay Kit and the quality of DNA fragments were determined on Fragment Analyzer 5200 (Agilent, USA).

Sixteen amplified DNA sample libraries were added to a single library pool with a combined mass of 1 µg and subsequently hybridized with above mentioned probes using HyperCap Target Enrichment Kit, HyperCap Bead Kit and SeqCap EZ probe pool (all Roche). The concentration and quality of captured libraries was determined on Fragment Analyzer 5200. The paired-end 2 × 75 bp sequencing was performed using Illumina NextSeq 500 Sequencer (NextSeq 0; Illumina San Diego, CA, USA).

Obtained NGS data were analyzed according to GATK pipelines recommended for data pre-processing for variant discovery and germline short variant discovery^82^. Hg38 was used as the reference genome. BWA 0.7.13 software package was used for alignment of reads^83^. Rest of analysis was performed using GATK4 4.1.2.0 software package^84^. Only polymorphisms with the depth of coverage higher than 50 reads were chosen for statistical analysis. Only polymorphisms which were determined in at least 50 samples were further statistically evaluated. In the selected samples the presence of polymorphisms in CD36 promotor region were confirmed by Sanger sequencing of two amplicons using the primer pairs 5′-GGTGTCTTTGCTTTAACCTTT-3′/5′-CTGGTCCCTAGCCTTTAATAG 3′ and 5′ ACCTAGCAACACTTAAGTACC-3′/5′-GAAGAATGGCTGTGGAATTTT-3′. The PCR were done in 20 µL reaction using the KAPA HiFi Hotstart ReadyMix (Roche, DE) and 0.3 µM primers with temperature profile of initial denaturation at 95 °C for 3 min and 35 cycles of denaturation at 98 °C for 20 s, annealing at 58 °C for 20 s and extension at 72 °C for 90 s with final extension of 5 min at 72 °C. The final PCR products were treated with ExoSAP-IT (ThermoFisher Scientific, USA) and sequenced at Eurofins Genomics (Germany) from both sites using the sequencing primers (Supplement Fig. 1).

### In silico studies of transcription factor binding sites

Analysis of transcription factor binding sites was performed using software PROMO v3.0.2, (which utilizes TRANSFAC v8.3)^85,86^. We used four homozygous patients and controls carrying homozygous variants of polymorphisms rs12667404, rs71518997 and rs2151916. The promotor regions of alternative CD36 Exon 1a (Gen-Bank: DA741325) were represented by 2000 bp upstream of transcription starting site and were loaded as the query sequence to search for potential binding sites. The prediction was carried out considering only the sites binding human transcription factors using the dissimilarity threshold parameter of 10% (90% similarity).

### In silico studies of miRNA binding sites

Sequences of 20 bases up- and downstream of the polymorphisms sites statistically significantly associated with a particular group were selected and tested as possible miRNA target against the most recent miRBase database (v22) using BLASTN search algorithm^87^ with E value cutoff at 10. The search was restricted to mature human miRNA sequences^51^. Additional analyses of the interactions by RNA22 v2 software^52^, with parameters set as Sensitivity of 45% vs Specificity of 78% and seed size of 7 allowing a maximum of 1 UN-paired base in the seed sequence, were further performed.

### Statistical analysis

Statistical analysis was performed separately for each group (i.e., HC, SCD, naMCI, aMCI, and DAT). The following variables were calculated for each subject and used in the statistical analysis: (1) the age at the time of the first examination (AGE); (2) the domain residual z-score at the first examination for each domain (i.e. AWM_SC, MEM_SC, EF_SC, LG_SC, and VS_SC); and (3) the slope of a simple ordinary least square regression line for the development of the domain residual z-score during the 2 years after the first examination with the time (calculated in days) elapsed since the first examination as an independent variable for each domain (i.e. AWM_DIF, MEM_DIF, EF_DIF, LG_DIF, and VS_DIF) if repeated examinations were available. The domain residual z-score values from the first and two subsequent examinations (examinations were performed at approximately 1-year intervals) were used to calculate AWM_DIF, MEM_DIF, EF_DIF, LG_DIF, and VS_DIF. Due to the application of this procedure, these variables express the average increase or decrease of the domain residual z-score per year.

All z-scores were calculated based on mean and standard deviation (SD) values of the HC group. Then, the effect of age was excluded using a simple ordinary least square regression with AGE as an independent variable calculated based on the values of the HC group. AWM_SC, MEM_SC, EF_SC, LG_SC and VS_SC values were calculated as residual values from the regression model. For all subsequent statistical analyses, the residual values were used instead of original z-scores. The same procedure was applied also to the z-scores obtained during follow-up examinations of patients, if available. Thus, the AWM_DIF, MEM_DIF, EF_DIF, LG_DIF and VS_DIF values are also calculated based on residual z-scores.

The association of the above variables with analyzed single nucleotide polymorphisms (SNPs) of the CD36 gene was tested using the Kruskal–Wallis test. Corrected significance level was calculated for each used variable to eliminate the effect of multiple comparisons. This was done through the iteration procedure performed independently in each group, with these steps performed during each random allocation: (1) the sequencing data were randomly assigned to patients within each group (sampling without replacement); (2) the association of each variable with all SNPs was tested using Kruskal–Wallis test; (3) for each variable, the lowest obtained P-value were then saved. After 1000 iterations, the process was terminated, and corrected significance level 0.05 was calculated for each variable as 5th percentile of obtained minimum P values. This corrected significance level was then used in deciding on the statistical significance of the association of the variable with SNPs. In the same way, the corrected significance level of 0.1 was determined to identify results approaching the significance level. These two thresholds were used in association testing.

To assess linkage disequilibrium for associated polymorphisms, the R^2^ measure was used. All statistical calculations were performed in R software^88^.

### Ethical approval and consent to participate

Informed consent was gained from all participants or their legal representatives and formal approval for the study was granted by ethics committee of St. Anne’s University Hospital Brno and Motol University Hospital (Praha).

### Consent for publication

All the individuals had signed consent forms for participating in this research project and use the obtained data in relevant publications.

## Supplementary Information

Supplementary Figure 1.

## Data Availability

The datasets generated during and/or analyzed during the current study are available from the corresponding author on reasonable request.

## Abbreviations

AD: Alzheimer’s disease
Aβ: Amyloid beta
aMCI: Amnestic mild cognitive impairment
ApoE: Apolipoprotein E
AWM: Attention and working memory
BNT: Boston Naming Test
CD36: Cluster of differentiation 36
CDT: Clock Drawing Test
DAT: Dementia of the Alzheimer type
DLB: Dementia with Lewy bodies
DNA: Deoxyribonucleic acid
DS-B: Backward Digit Span Subtests
DS-F: Forward Digit Span Subtests
EDTA: Ethylenediaminetetraacetic acid
ECR-FR: Enhanced Cued Recall free recall
ECR-TR: Enhanced Cued Recall total recall
EF: Executive function
EOAD: Early onset Alzheimer’s disease
FTLD: Frontotemporal lobar degeneration
HC: Healthy control
IL-18: Interleukin 18
IL-1α: Interleukin 1α
IL-1β: Interleukin 1β
LG: Language
LOAD: Late-onset Alzheimer’s disease
MCI: Mild cognitive impairment
MEM: Memory
miRNA: Micro RNA
MMSE: Mini-Mental State Examination
MRI: Magnetic resonance imaging
naMCI: Non-amnestic mild cognitive impairment
NGS: Next generation sequencing
NLRP3: NOD-like receptor 3
PDD: Parkinson's disease dementia
P-VF: Phonemic verbal fluency
RAVLT: Rey Auditory Verbal Learning Test
ROCFT: Rey–Osterrieth Complex Figure Test
SCD: Subjective cognitive decline
SNP’s: Single nucleotide polymorphisms
S-VF: Semantic verbal fluency test
TF: Transcription factor
TFBSs: Transcription factor binding sites
TLR: Toll-like receptor
TMT: Trail Making Test
VS: Visuospatial function
