# Supplementary material for: Six genetically linked mutations in the CD36 gene significantly delay the onset of Alzheimer's disease
Source: Sci Rep. 2022 Jun 29;12:10994. doi: 10.1038/s41598-022-15299-z (PMC9243110; doi:10.1038/s41598-022-15299-z)
Supplement: Supplementary file 1 — Supplementary Figure 1. [file 41598_2022_15299_MOESM1_ESM.pdf]

```
                     10        20        30        40        50        60        70        80        90       100       110       120
             ....|....|....|....|....|....|....|....|....|....|....|....|....|....|....|....|....|....|....|....|....|....|....|....|
NC_000007.14 AGGCCCCATACCCAGCCACATGGGGGACTTTCCCACCTTCAGGTGGGGGGACCACCCCTGCATCTCCTCTCTGCTGAAAGCTGTTCCATCACTCAATAAAATTATTTTCTGTCTTCCTCA
12           ....................................................................................................G...................
19           ....................................................................................................G...................
16           ........................................................................................................................
36           .......................................................T................................................................
40           .......................................................T................................................................
269          .......................................................T................................................................
324          .......................................................T................................................................
131          .......................................................T................................................................
154          ........................................................................................................................
163          ........................................................................................................................

                    130       140       150       160       170       180       190       200       210       220       230       240
             ....|....|....|....|....|....|....|....|....|....|....|....|....|....|....|....|....|....|....|....|....|....|....|....|
NC_000007.14 CCCTTCAATATTCACCATATCCTCATTCTTCTGGGGCACGAGACAAGAGCTTGGGAAACGCTGAATGCTGGTATAAGCTGGGACATGCCAGTGTGGCCAAGAGAGGCCCAGGTGGGGCAT
12           ........................................................................................................................
19           ........................................................................................................................
16           ........................................................................................................................
36           ........................................................................................................................
40           ........................................................................................................................
269          ........................................................................................................................
324          ........................................................................................................................
131          ........................................................................................................................
154          ........................................................................................................................
163          ........................................................................................................................

                    250       260       270       280       290       300       310       320       330       340       350       360
             ....|....|....|....|....|....|....|....|....|....|....|....|....|....|....|....|....|....|....|....|....|....|....|....|
NC_000007.14 GGCTTGCCGAGGGTCCCCGGCTTGCAAAGAGACCATGAAGAAAAATCTTACCTTACTAGCATTGCTGAAGGAGAACAAAGAAGAATAATGGCATCCACCAAAAATCAAAGCAATACTGTC
12           ........................................................................................................................
19           ........................................................................................................................
16           ........................................................................................................................
36           ........................................................................................................................
40           ........................................................................................................................
269          ........................................................................................................................
324          ........................................................................................................................
131          ........................................................................................................................
154          ........................................................................................................................
163          ........................................................................................................................

                    370       380       390       400       410       420       430       440       450       460       470       480
             ....|....|....|....|....|....|....|....|....|....|....|....|....|....|....|....|....|....|....|....|....|....|....|....|
NC_000007.14 TCAAACATTACAGCAGAACTTGGAGAAAGGTGTTAAAAAGAGATAAAAAGTGGACACAGAAAAAAGAGCAAAACAAAGCAATATTCTAGTTACATTTTTAAAAATAATCATTAGGATGAT
12           ......................................................A.T...............................................................
19           ......................................................A.T...............................................................
16           ......................................................A.T...............................................................
36           ......................................................A.T...............................................................
40           ......................................................A.T...............................................................
269          ......................................................A.T...............................................................
324          ......................................................A.T...............................................................
131          ......................................................A.T...............................................................
154          ......................................................A.T...............................................................
163          ......................................................A.T...............................................................
```

```
                          490       500       510       520       530       540       550       560       570       580       590       600
                  ....|....|....|....|....|....|....|....|....|....|....|....|....|....|....|....|....|....|....|....|....|....|....|....|
NC_000007.14      TTAGAACAAGTGTTTGTAACTCTGGGGGTCTCTGAACCCTCATTTGTAGGACTAGATGTCTGCAGTGAGAAAATAAGAAAGGAAAGAGAAGAAATGTATTCTACACAGAAGTAGGGGAAA
12                ........................................................................................................................
19                ........................................................................................................................
16                ........................................................................................................................
36                ........................................................................................................................
40                ........................................................................................................................
269               ........................................................................................................................
324               ........................................................................................................................
131               ........................................................................................................................
154               ........................................................................................................................
163               ........................................................................................................................

                          610       620       630       640       650       660       670       680       690       700       710       720
                  ....|....|....|....|....|....|....|....|....|....|....|....|....|....|....|....|....|....|....|....|....|....|....|....|
NC_000007.14      GTTCCTTAAACTATGAGTCACAGCTATTTATATCCGAACAAATGTGCATTTCCTATTAGTCCAGGAGATTCTGTGGTCTTCTACCCATGCATTTGTATAAAGAATTGTCCTCTGTGTATA
12                ........................................................................................................................
19                ........................................................................................................................
16                ........................................................................................................................
36                ........................................................................................................................
40                ........................................................................................................................
269               ........................................................................................................................
324               ........................................................................................................................
131               ........................................................................................................................
154               ........................................................................................................................
163               ........................................................................................................................

                          730       740       750       760       770       780       790       800       810       820       830       840
                  ....|....|....|....|....|....|....|....|....|....|....|....|....|....|....|....|....|....|....|....|....|....|....|....|
NC_000007.14      AGCTGCATCTCTAGAATATGAACTCCTTGAAACTACCTAGCAACACTTAAGTACCTAGACCAATTTGGACAGACAGCATTATTGGGTTGTCTTGGTAAATATTGGTAACTTCAATAACCA
12                ........................................................................................................................
19                ........................................................................................................................
16                ........................................................................................................................
36                ........................................................................................................................
40                ........................................................................................................................
269               ........................................................................................................................
324               ........................................................................................................................
131               ........................................................................................................................
154               ........................................................................................................................
163               ........................................................................................................................

                          850       860       870       880       890       900       910       920       930       940       950       960
                  ....|....|....|....|....|....|....|....|....|....|....|....|....|....|....|....|....|....|....|....|....|....|....|....|
NC_000007.14      AGAATGCTCTAAAATCTATAGCTAGACAACAGGCTATCTCTGAACTAAACTTTAGACAACAGTCCTTGGTAGACTTTTTTTTTTTTTTTACTCTATTATGTAAAAGTAGAGACTTATCTA
12                ........................................................................................................................
19                ........................................................................................................................
16                ........................................................................................................................
36                ...............................................C........................................................................
40                ...............................................C........................................................................
269               ...............................................C........................................................................
324               ...............................................C........................................................................
131               ...............................................C........................................................................
154               ........................................................................................................................
163               ........................................................................................................................
```

```
                     970       980       990      1000      1010      1020      1030      1040      1050      1060      1070      1080
              ....|....|....|....|....|....|....|....|....|....|....|....|....|....|....|....|....|....|....|....|....|....|....|....|
NC_000007.14  GTAATAATTCTAGCTGGTTCATAAATCTCTGATACCATTAATTTCTCTATTGAAGATCTCATTGTTAGTATTTATTATACCAATCTTCTGTTCTAAAGAGATAAAAAATAACTAAATTTT
12            ........................................................................................................................
19            ........................................................................................................................
16            ........................................................................................................................
36            ........................................................................................................................
40            ........................................................................................................................
269           ........................................................................................................................
324           ........................................................................................................................
131           ........................................................................................................................
154           ........................................................................................................................
163           ........................................................................................................................

                    1090      1100      1110      1120      1130      1140      1150      1160      1170      1180      1190      1200
              ....|....|....|....|....|....|....|....|....|....|....|....|....|....|....|....|....|....|....|....|....|....|....|....|
NC_000007.14  GGACTTCTATTAAAGGCTAGGGACCAGCTCTAGAATGAATGTTCTCGACCAGGGTGACTGTCCACTTCATGAAAGTAGACTAAAAATACCAAACTCTCATATATACACAATAATATATAT
12            ..............................G.........................................................................................
19            ..............................G.........................................................................................
16            ..............................G.........................................................................................
36            ........................................................................................................................
40            ........................................................................................................................
269           ........................................................................................................................
324           ........................................................................................................................
131           ........................................................................................................................
154           ..............................G.........................................................................................
163           ..............................G.........................................................................................

                    1210      1220      1230      1240      1250      1260      1270      1280      1290      1300      1310      1320
              ....|....|....|....|....|....|....|....|....|....|....|....|....|....|....|....|....|....|....|....|....|....|....|....|
NC_000007.14  CAAAGTATTTGAAGTACATTATAGAAATCCTCACAGTAGGGGTCACATTTTCTTCCACTTTGTGGCAGAGTTCTGAGCACATAGTGACCATGTGATAAATATTAATTCAGTATGTGAGTG
12            ........................................................................................................................
19            ........................................................................................................................
16            ........................................................................................................................
36            ..............................................................--........................................................
40            ..............................................................--........................................................
269           ..............................................................--........................................................
324           ..............................................................--........................................................
131           ..............................................................--........................................................
154           ........................................................................................................................
163           ........................................................................................................................

                    1330      1340      1350      1360      1370      1380      1390      1400      1410      1420      1430      1440
              ....|....|....|....|....|....|....|....|....|....|....|....|....|....|....|....|....|....|....|....|....|....|....|....|
NC_000007.14  TATGAGGATGTATGTCATCAATATTCTTTGACTGCCTTAAGTGTTCTGGGAAAAAGCCATATTTTTGGTCTCATTTAGCATATACTGTAATT----CTTATTTTTAGGGTGTATTTATTT
12            ............................................................................................AATA........................
19            ............................................................................................AATA........................
16            ............................................................................................AATA........................
36            ............................................................................................AATA........................
40            ............................................................................................AATA........................
269           ............................................................................................AATA........................
324           ............................................................................................AATA........................
131           ............................................................................................AATA........................
154           ............................................................................................AATA........................
163           ............................................................................................AATA........................
```

```
                 1450      1460      1470      1480      1490      1500      1510      1520      1530      1540      1550      1560
             ....|....|....|....|....|....|....|....|....|....|....|....|....|....|....|....|....|....|....|....|....|....|....|....|
NC_000007.14 TTGTTTTAATTTATTTGATTTGTGTTGCATATACAATATTGTACCCATCAAATATTGCTTAATTCTTTCATATTGCAAAATAAACAGCTATTATAAGCCTGAACTTGTTAGTCTTGCTGG
12           ........................................................................................................................
19           ........................................................................................................................
16           ........................................................................................................................
36           ........................................................................................................................
40           ........................................................................................................................
269          ........................................................................................................................
324          ........................................................................................................................
131          ........................................................................................................................
154          ........................................................................................................................
163          ........................................................................................................................

                 1570      1580      1590      1600      1610      1620      1630      1640      1650      1660      1670      1680
             ....|....|....|....|....|....|....|....|....|....|....|....|....|....|....|....|....|....|....|....|....|....|....|....|
NC_000007.14 GCCCTGCCCAAGGTTGCCCTCATCTCCAGCTTTCCACAAACTGGAATATTCACTGATGCTTTGTTTCTTCTCCCAGACCAGGATACATGTTGTTATGTGGTTCCTAGGAGGACTGTAAAG
12           ........................................................................................................................
19           ........................................................................................................................
16           ........................................................................................................................
36           ........................................................................................................................
40           ........................................................................................................................
269          ........................................................................................................................
324          ........................................................................................................................
131          ........................................................................................................................
154          ........................................................................................................................
163          ........................................................................................................................

                 1690      1700      1710      1720      1730      1740      1750      1760      1770      1780      1790      1800
             ....|....|....|....|....|....|....|....|....|....|....|....|....|....|....|....|....|....|....|....|....|....|....|....|
NC_000007.14 TCCTCTAACAAAGACAAAGGATGGAATGAATCAAGGGAATTACTATGAGAAAGTAGGGATATCCCTGGAGAGGGGACTGTTTCTGTGTGCTTTTAGACATCACAGAGTAATTGTTTCTTT
12           ........................................................................................................................
19           ........................................................................................................................
16           ........................................................................................................................
36           ........................................................................................................................
40           ........................................................................................................................
269          ........................................................................................................................
324          ........................................................................................................................
131          ........................................................................................................................
154          ........................................................................................................................
163          ........................................................................................................................

                 1810      1820      1830      1840      1850      1860      1870      1880      1890      1900      1910      1920
             ....|....|....|....|....|....|....|....|....|....|....|....|....|....|....|....|....|....|....|....|....|....|....|....|
NC_000007.14 TTTTAAAGGAAGGAACCAGGGTGAAGAGCAGCTCATTTTTAAGCTGGAGAGGTTAACAAACACTGGCTGCAAGATTTACTTTGGTTTAGGAATACCACTGTCTCACAGGAGTGTCAATCA
12           ........................................................................................................................
19           ........................................................................................................................
16           ........................................................................................................................
36           ........................................................................................................................
40           ........................................................................................................................
269          ........................................................................................................................
324          ........................................................................................................................
131          ........................................................................................................................
154          ........................................................................................................................
163          ........................................................................................................................
```

```
                       1930      1940      1950      1960      1970      1980      1990      2000
                   ....|....|....|....|....|....|....|....|....|....|....|....|....|....|....|....|....|
NC_000007.14       AAATTCCACAGCCATTCTTCGACACTAGGAGGTGGGACTGACTGATGTACAGCAGTGATTTGACCCAGCACTTGGGGCAAACACA
12                 .....................................................................................
19                 .....................................................................................
16                 .....................................................................................
36                 ...........................................................C.........................
40                 ...........................................................C.........................
269                ...........................................................C.........................
324                ...........................................................C.........................
131                ...........................................................C.........................
154                .....................................................................................
163                .....................................................................................
```
